# Air Pollutants, Climate, and the Prevalence of Pediatric Asthma in Urban Areas of China

**DOI:** 10.1155/2016/2935163

**Published:** 2016-07-31

**Authors:** Juanjuan Zhang, Jihong Dai, Li Yan, Wenlong Fu, Jing Yi, Yuzhi Chen, Chuanhe Liu, Dongqun Xu, Qiang Wang

**Affiliations:** ^1^Center of Respiratory Disorders, Children's Hospital, Chongqing Medical University, Chongqing 400014, China; ^2^Ministry of Education Key Laboratory of Child Development and Disorders, Chongqing 400014, China; ^3^Department of Medical Statistics, Chongqing Medical University, Chongqing 400046, China; ^4^Center for Asthma Prevalence and Education, Capital Institute of Pediatrics, Beijing 100020, China; ^5^Institute of Environmental Health and Related Products Safety, Chinese Center for Disease Control and Prevention, Beijing 100021, China

## Abstract

*Background*. Prevalence of childhood asthma varies significantly among regions, while its reasons are not clear yet with only a few studies reporting relevant causes for this variation.* Objective*. To investigate the potential role of city-average levels of air pollutants and climatic factors in order to distinguish differences in asthma prevalence in China and explain their reasons.* Methods*. Data pertaining to 10,777 asthmatic patients were obtained from the third nationwide survey of childhood asthma in China's urban areas. Annual mean concentrations of air pollutants and other climatic factors were obtained for the same period from several government departments. Data analysis was implemented with descriptive statistics, Pearson correlation coefficient, and multiple regression analysis.* Results*. Pearson correlation analysis showed that the situation of childhood asthma was strongly linked with SO_2_, relative humidity, and hours of sunshine (*p* < 0.05). Multiple regression analysis indicated that, among the predictor variables in the final step, SO_2_ was found to be the most powerful predictor variable amongst all (*β* = −19.572,* p* < 0.05). Furthermore, results had shown that hours of sunshine (*β* = −0.014,* p* < 0.05) was a significant component summary predictor variable.* Conclusion*. The findings of this study do not suggest that air pollutants or climate, at least in terms of children, plays a major role in explaining regional differences in asthma prevalence in China.

## 1. Introduction

Asthma is one of the most chronic inflammatory disorders characterized by recurrent attacks of wheezing, breathlessness, cough, and/or chest tightness, which vary in severity and frequency from person to person. The prevalence of allergic airway diseases, such as asthma and rhinitis, has been increasingly common to epidemic proportions worldwide. About 334 million people worldwide are now suffering from asthma [1]. And China is one of the most asthma-afflicted countries, and its population of asthmatics is estimated to be approximately 30 million, including 10 million children [2].

And, more importantly, there are significantly striking differences in asthma symptoms worldwide [3, 4]. The ISAAC Phase Three has surveyed 800,000 children aged from 13 to 14 years in 233 collaborating centers in 98 countries and found that the prevalence of asthma symptoms ranged from 0.8% to 32.6% in countries [4, 5]. And the Chinese National Cooperative Group on Children Asthma has conducted a sample survey from 1988 to 1990 [6] to assess the prevalence of asthma in children aged 0–14 years in China. In this survey, the reported prevalence of asthma (defined as having experienced an asthma attack in the past two years) ranged from 0.09 to 2.60%, with an average of 0.91%. In 2000 [6], after a 10-year interval, the same survey was conducted again. The preliminary findings indicated that the prevalence of asthma had increased to 0.52–3.34% in city-dwelling children, with the national average being 1.54%. In 2010 [7], 20 years after the original survey, the same methodology found that the prevalence of childhood asthma was estimated at 0.42–5.73%, with the national average being 2.32%. These results highlight two key conclusions: first, the number of pediatric asthma patients has risen over time; second, the prevalence of childhood asthma in China varies among regions.

A large number of studies have confirmed that respiratory diseases are related to the physical characteristics of the living area: asthma has multifactorial etiology, including exogenous factors like air pollution and climate. The link between urban air pollution, climatic factors, and asthma is stably established on the individual level. However, there are merely a few analyses developing a comparative approach of differences in asthma morbidity among places, such as cities. In addition, levels of outdoor air pollutants have been associated with asthma incidence but not clearly with asthma prevalence at the population level [8]. Under this circumstance, this paper investigated the regional disparities' correlation with air pollutants and climatic factors in different cities in China, with cities as the objects of the study. This is in contrast to many epidemiological study designs where information is available at the individual level. The result could offer novel insights into the impact of air pollutants and climate on asthma prevalence.

## 2. Material and Methods

### 2.1. Study Design and Criteria

The data of asthma, air pollutants, and other climatic factors were obtained from published journals or government departments. Asthma definition in this survey was based on the Global Initiative for Asthma, Chinese Guideline of Pediatric Asthma published in 2008 [9]. In the questionnaire, the primary inclusion criterion for asthma patients was a subject with definite wheezing three times and more. Stratified random sampling was to be adopted in this survey, and it was uniformly carried out by the Chinese National Cooperative Group on Children Asthma.

### 2.2. Asthma Data

Asthma data in this study were collected from the open-access Chinese Journal of Pediatrics [9]. The Chinese National Cooperative Group on Children Asthma organized the third nationwide survey of childhood asthma in urban areas of China, which included 463,982 children and identified 13,992 asthmatic patients aged 0–14 years. The national survey was conducted in 43 cities across the country, and children's demographic characteristics were available. The selected data of asthma prevalence were defined as children who had experienced at least one asthma attack in the last two years (2009-2010), which contained 10,777 asthmatic patients. Data pertaining to the clinical and demographic characteristics of asthmatic patients in the 43 specific cities were not described as they were not made available by the third national survey.

Approval for the use of the data was obtained from the National Cooperative Group on Children Asthma. And informed consent was obtained from the next of kin, caretakers, or guardians on behalf of children. The consent on behalf of the children enrolled was written. The study design was approved by the ethics committee of the National Cooperative Group on Children Asthma.

### 2.3. Air Pollution Data

Air pollution data included the annual (from 2009 to 2010) average values for particulate matter less than or equal to 10 *µ*m PM10 (*µ*g/m^3^), sulfur dioxide SO_2_ (*µ*g/m^3^), and nitrogen dioxide NO_2_ (*µ*g/m^3^). All the data were obtained from the State Statistical Bureau Network which collected annual average concentrations of air pollutants through automated fixed-site monitoring stations. The data were regularly recorded from January 1, 2009, to December 31, 2010. We collected and calculated the 2-year average (from 2009 to 2010) values of these variables, yet numbers of air quality monitoring stations in 43 cities were not available.

The World Health Organization Air Quality Guidelines for annual mean concentrations of PM10 and NO_2_ and for 24-hour mean concentrations of SO_2_ were 20 *µ*g/m^3^, 40 *µ*g/m^3^, and 20 *µ*g/m^3^, respectively [10].

### 2.4. Meteorological Data

Meteorological data included the annual average values for temperatures (°C), relative humidity (%), precipitation (mm), and hours of sunshine (h). Obtained from the State Statistical Bureau Network and the China Meteorological Administration, these data were regularly recorded from January 1, 2009, to December 31, 2011, as well. We collected and calculated the 2-year averaged (from 2009 to 2010) value of these variables. The numbers of weather monitoring stations in 43 cities were also not available.

### 2.5. Statistical Methods

Descriptive statistics were used to summarize the general characteristics of the data included in this study. The Shapiro-Wilk test revealed a normal distribution of tested variables. The mean and standard deviation (SD) of each of the variables were then calculated. Associations were assessed with Pearson correlation coefficient and multiple regression analysis. Prevalence of asthma was the dependent variable, and independent variables were air pollutants (PM10, SO_2_, and NO_2_) and climatic factors (relative humidity, air temperature, precipitation, and hours of sunshine). Statistical significance was set at *p* < 0.05, and all statistical analyses were performed with SPSS software version 20.0.

## 3. Results

### 3.1. Baseline Characteristics of Patients in the Third National Survey on Childhood Asthma

Table 1 shows the baseline characteristics of the patients from the third nationwide study of childhood asthma in urban areas of China [7].

The national prevalence of asthma in male and female children was 3.51% and 2.29%, respectively. The prevalence of asthma was significantly higher in preschool children (*n* = 1,127, 4.15%) than in school-age children (*n* = 8,429, 2.82%) and infants (*n* = 4,026, 1.77%). Within the total survey cohort, 12,997 (3.00%) patients of Han descent, 87 (2.73%) patients of Manchu descent, and 157 (2.39%) patients of Hui descent were asthmatic. In the asthmatic patients, respiratory tract infection (*n* = 12,299, 87.9%) and changes in the weather conditions (*n* = 7,204, 51.5%) were the most frequent triggers of asthma exacerbation. Common clinical manifestations of asthma were cough (*n* = 12,771, 91.3%) and wheezing (*n* = 10,659, 76.2%). A family history of allergies was reported in 45.2% of patients (*n* = 6,321), and a personal history of allergies was reported in 72.5% of patients (*n* = 10,143). Regarding the frequency of asthma attacks each year, 87.7% of patients reported 1–5 asthma attacks, 10.1% reported 6–10 attacks, and only 2.2% reported more than 10 attacks. To treat asthmatic children, bronchodilators were used in 71.4% of cases (*n* = 9,986), inhaled corticosteroids in 58.7% (*n* = 8,209), and antibiotics in 75.1% (*n* = 10,504).

### 3.2. Asthma Prevalence, Air Pollutants, and Climatic Factors in 43 Cities

Table 2 is the summary of the prevalence of childhood asthma provided by the third national survey and the annual mean concentrations of air pollutants (PM10, SO_2_, and NO_2_), together with the annual mean levels of climatic factors (relative humidity, air temperature, precipitation, and hours of sunshine) for each of the 43 cities. There were statistically significant differences in asthma prevalence, air pollutants, and climatic factors between cities (Table 3). The most prevalent childhood asthma was found in Shanghai (5.73%) and the least was in Lasa (0.42%). The air index is listed as follows: the concentration of PM10 ranged from 0.024 to 0.153 mg/m^3^ and averaged 0.091 mg/m^3^, with a standard deviation of 0.026 mg/m^3^; the concentration of SO_2_ ranged from 0.007 to 0.091 mg/m^3^ and averaged 0.042 mg/m^3^, with a standard deviation of 0.018 mg/m^3^; the concentration of NO_2_ ranged from 0.016 to 0.160 mg/m^3^ and averaged 0.042 mg/m^3^, with a standard deviation of 0.015 mg/m^3^; relative humidity ranged from 32.000 to 81.000% and averaged 66.008%, with a standard deviation of 10.366%; the air temperatures over the course of the study ranged from 4.75 to 24.440°C and averaged 15.125°C, with a standard deviation of 4.944°C; precipitation ranged from 15.75 to 211.39 mm and averaged 82.92 mm, with a standard deviation of 46.516 mm; the duration of sunshine ranged from 67.880 to 265.810 h and averaged 166.08 h, with a standard deviation of 43.524 h.

### 3.3. Correlations between Asthma Prevalence, Air Pollutants, and Climatic Factors in 43 Cities

The results of the Pearson correlation coefficient and multiple regression analysis describing the relationship between asthma prevalence and risk factors (air pollutants and climatic factors) are shown in Tables 4 and 5. According to Pearson correlation analysis (Table 4), there were significant associations between asthma prevalence and several factors including SO_2_ (*r* = −0.323, *p* < 0.05), relative humidity (*r* = −0.351, *p* < 0.05), and hours of sunshine (*r* = −0.476, *p* < 0.05). As can be seen, these correlations between PM10, NO_2_, air temperature, precipitation, and asthma prevalence were not statistically significant. Therefore, the multiple linear regression analysis with a dependent variable of asthma prevalence and independent variables of SO_2_, relative humidity, and hours of sunshine was used to predict asthma prevalence. The prediction model was statistically significant (*F* = 5.573, *p* < 0.01) and accounted for approximately 30.3% of the variance of asthma prevalence (*R* = 0.608, adjusted *R* ^2^ = 0.370). Multiple regression analysis indicated that, among the predictor variables, SO_2_ was found to be the most powerful predictor variable amongst all (*β* = −19.572,* p* < 0.05). Furthermore, results had shown that hours of sunshine (*β* = −0.014,* p* < 0.05) was a significant component summary predictor variable. However, relative humidity was not significantly predicted to be the outcome measure.

## 4. Discussion

The aim of this study was to investigate the potential role of city-average air-pollutant levels and climatic factors in explaining regional differences in asthma prevalence in China. Variables for significant correlation coefficients were also obtained in the multiple regression analysis. They were the mean SO_2_ and hours of sunshine. Before the discussion of the findings, it is important to address methodological aspects of this study. This paper does not focus on the individuals themselves but cities, which is in contrast to many epidemiological study designs where information is available at the individual level. In addition, our data was available at annual mean level rather than daily. The size of the study was large, and although large-scale researches were accomplished by ISAAC in reference to asthma for air pollution [5, 11] and climate [5, 12], their 43 cities in China made for acceptable discrimination.

A growing body of research on time trends is devoted to the relationship between allergic respiratory diseases including asthma and environmental factors, climate conditions and air pollutants, but results on effects of these variables on asthma are still unclear and current knowledge is provided by epidemiological and experimental studies.

The adverse effect of air pollution on respiratory health has been well established in the literature [13, 14], but our results are not consistent with this evidence. The concentration of pollutants such as PM10 and NO_2_ no longer provides a statistical explanation for variations in asthma prevalence across urban units. Interestingly, the relationship between childhood asthma and SO_2_ was consistently negative. And surprisingly, these annual mean levels of PM10 (0.091 mg/m^3^), NO_2_ (0.042 mg/m^3^), and SO_2_ (0.042 mg/m^3^) detected during the sampling period did not conform with the data from World Health Organization Air Quality Guidelines [10]. This result is consistent with previous intercommunity studies [10, 15–18], including ones of the ISAAC [5, 15, 16] which did not observe positive associations between ambient air pollutants and asthma prevalence. However, a study of individual level by ISAAC found a positive association between asthma prevalence and proximity to traffic pollution [19]. The disparities between these findings and those of within-community studies of individuals exposed to air pollutants remain to be further explained. This is compatible with the hypothesis that outdoor levels of air pollutants have been associated with asthma incidence but not with asthma prevalence at the population level.

The influence of short-term climate change on asthma exacerbation is well established, but its long-term influence on disease occurrence is little studied.

Regarding the effect of climatic variables, a large number of studies have confirmed that temperature is related to asthma [12, 20–31]. However, for our study, there has not been shown any statistically significant association between annual mean temperature and asthma prevalence, which are similar to some established in extraurban studies [12, 22]. Our results are different, because asthma prevalence was the object of study and not asthma incidence or asthma symptoms in the literature [12, 21, 25], and climatic parameters considered in the literature vary: annual mean minimum or maximum temperature [23], annual mean or seasonal mean temperature [12, 21, 26, 27], mean temperature for the coldest or hottest months [22], daily temperature [1, 17, 24, 25], and monthly temperature [28–31]. Thus, a direct comparison of results is not possible.

In the present study, annual mean relative humidity was not related to asthma prevalence. Indeed, relative humidity has been associated with the prevalence of wheezing [26, 29], symptoms, diagnosis, and incidence of asthma [5, 24, 26–28, 32, 33]. But a few studies failed to show significant associations [12, 30, 31], most likely due to different parameters. As mentioned above, our data were available on annual mean relative humidity, not including minimum or maximum relative humidity and other parameters. And our results indicate that hours of sunshine is a protective factor with the prevalence of asthma in the cities studied, which is consistent with results reported by recent epidemiological studies [11, 28–30, 34]. In addition, a recent analysis of ISAAC data supports the argument that there exists a negative relationship between the prevalence of asthma and the mean annual sunny hours at a community level within centers [35]. Indirectly, the results could support the hypothesis of the protective effect of vitamin D in the prevalence of asthma in children [35].

These few studies analyzing associations of asthma with precipitation [28, 30, 31, 36] showed mixed results. There is no confirmation in the literature that the association between climate and asthma prevalence reflects a causal relationship or that correlations are a result of indirect relationships and linked to other factors like air pollution levels. Little is known about the long-term influence of climate on asthma prevalence, but the findings from our study do not suggest that climate, at least in children, plays a major role.

The reasons for the inconsistencies among our study and previous studies are possibly regional discrepancies such as geographical environment, economic development, and density of the local populations. Additionally, study design, analyzing method, subgroups of children, and different parameters may also contribute to the controversies. Thus, a direct comparison of results is not accurate.

On the other hand, there are still several limitations in this study. First, modeled estimates of air pollutants and climatic factors at city level are imprecise and incomplete in terms of personal exposure to ambient air pollutants. Second, collinearity between variables and the time series impeded the evaluation of particular pollutants or climatic factors that had a direct adverse effect on the prevalence of asthma. Third, other factors such as the indoor environment [37], economic development [38, 39], dietary factors [40], pollen levels [41], and infections [42], which were not accounted for in our analysis, could have precipitated the regional differences in asthma prevalence. Fourth, our data were given on annual basis rather than monthly or daily. Under this circumstance, any short-term effect of short duration weather and air pollution changes could be lost, but not of changes for longer duration. Fifth, there might be subjects who were not new onset asthmatic patients. Therefore the two-year data could not precisely reflect the relationship between these factors and asthma prevalence. Finally, the data from the third national survey did not provide the clinical and demographic characteristics of the asthmatic patients in the specific cities and the precise time of asthma prevalence we used for our analyses. The limited access to this information hindered our pace to dig in the relationship between climatic and air quality factors and the clinical presentation of asthma in each city.

## 5. Conclusion

To conclude, our study showed associations between long-term exposure to urban air pollution and climate conditions, using Pearson correlation coefficient and multiple regression analysis capturing variations within communities, and asthma. First, the study shows the surprising results for the contribution of pollution factors. In contrast to reports from within-community studies of individuals exposed to traffic pollution, we found no evidence of a positive relation between air pollution and asthma prevalence. Meanwhile, our results suggest that climate may affect the prevalence of asthma, yet climate does not play a major role, at least in terms of children.

## Figures and Tables

**Table 1:** Demographic characteristics of the children surveyed in the 3rd national childhood asthma survey^a^.

	Full survey (*n*, total)	Asthmatic patients (*n* [% of full-survey population])^c^
*Number of patients*	463,982	13,992 (3.02%)
*Gender*		
Male	241,811	8,495 (3.51%)
Female	222,160	5,089 (2.29%)
*Age*		
Infant (0–2 years)	63,717	1,127 (1.77%)
Preschool (3–5 years)	97,075	4,026 (4.15%)
School age (6–14 years)	303,245	8,429 (2.82%)
*Race*		
Han	433,951	12,997 (3.00%)
Manchus	3,189	87 (2.73%)
Zhuang	4,136	109 (2.64%)
Hui	6,574	157 (2.39%)
Mongolian	1,520	29 (1.91%)
Uygur	1,781	14 (0.79%)
Tibetan	4,346	22 (0.51%)
*Inducing factors*		*(n [% of total asthmatic children])*
Respiratory infection	—	12,299 (87.9%)
Climate change	—	7,204 (51.5%)
Exercise	—	3,055 (21.8%)
*Clinical features*	—	
Cough	—	12,771 (91.3%)
Wheezing	—	10,659 (76.2%)
*Asthma attacks per year* ^b^		
1–5	—	10,682 (87.7%)
6–10	—	1,227 (10.1%)
>10	—	269 (2.2%)
*Allergy history*		
Personal allergies	—	10,143 (72.5%)
Allergic rhinitis	—	7,010 (50.1%)
Eczema/atopic dermatitis	—	4,147 (29,6%)
Urticaria	—	2,683 (19,2%)
Food allergies	—	2,079 (14.9%)
Family history of allergies	—	6,321 (45.2%)
Family history of asthma	—	2,924 (20.9%)
*Therapy*		
Bronchodilators	—	9,986 (71.4%)
Steroids	—	8,209 (58.7%)
Leukotriene antagonist	—	4,873 (34.8%)
Antiallergic agents	—	6,352 (45.4%)
Antibiotics	—	10,504 (75.1%)

(a) Data from the Chinese Journal of Pediatrics: third-nationwide survey of childhood asthma in urban areas of China, 2013.

(b) Data regarding the frequency of asthma attacks was only available for 12,178 (87%) patients in the asthmatic cohort.

(c) Data pertaining to the prevalence, age, gender, and race of the asthmatic patients is presented as the percentage of the corresponding population in the full survey. For example, 3.51% of all males surveyed met the criteria for an asthma diagnosis.

**Table 2:** Levels of childhood asthma prevalence, air pollutants, and climatic factors in Chinese cities.

City	Asthma prevalence (%)	Air pollutants			Climatic factors			
		PM10 (mg/m^3^)	SO_2_ (mg/m^3^)	NO_2_ (mg/m^3^)	Relative humidity (%)	Air temperature (°C)	Precipitation (mm)	Hours of sunshine (h)
Beijing	344 (2.55)	0.121	0.033	0.055	51.0	12.93	41.80	203.95
Tianjin	273 (2.14)	0.099	0.055	0.043	58.5	12.53	38.40	185.24
Shijiazhuang	121 (1.23)	0.101	0.050	0.038	56.0	14.20	47.16	193.88
Taiyuan	124 (1.22)	0.098	0.072	0.021	53.0	11.18	41.74	202.58
Baotou	102 (0.90)	0.105	0.062	0.037	46.5	8.15	24.14	243.30
Shenyang	148 (1.62)	0.106	0.059	0.036	69.0	7.47	70.60	205.64
Changchun	187 (1.56)	0.087	0.032	0.044	63.0	5.63	56.64	196.35
Harbin	109 (1.03)	0.101	0.046	0.051	67.5	4.75	46.89	181.71
Shanghai	755 (5.73)	0.080	0.032	0.052	69.5	17.30	100.76	139.29
Nanjing	206 (1.60)	0.107	0.036	0.047	71.5	16.31	110.91	156.77
Hangzhou	496 (3.57)	0.098	0.038	0.054	71.5	17.60	132.58	141.63
Hefei	483 (5.18)	0.113	0.022	0.029	72.5	16.57	94.53	152.51
Fuzhou	479 (4.08)	0.069	0.012	0.036	72.0	20.53	124.13	128.80
Nanchang	240 (2.33)	0.083	0.055	0.040	71.5	18.65	145.37	156.43
Jinan	275 (2.02)	0.120	0.048	0.026	54.0	14.55	63.45	173.65
Zhengzhou	329 (3.19)	0.105	0.053	0.046	58.5	15.55	56.78	149.75
Wuhan	144 (2.12)	0.107	0.043	0.056	74.0	17.24	104.00	138.95
Changsha	229 (1.80)	0.088	0.040	0.044	73.0	18.36	118.46	146.52
Guangzhou	131 (1.12)	0.070	0.036	0.055	71.5	22.77	159.43	131.49
Nanning	275 (2.01)	0.060	0.030	0.029	76.0	22.01	97.50	139.80
Haikou	300 (2.90)	0.039	0.007	0.016	81.0	24.44	211.39	153.52
Chongqing	424 (3.64)	0.104	0.051	0.038	79.0	18.82	93.48	77.27
Chengdu	413 (3.42)	0.108	0.035	0.053	76.5	16.42	69.21	67.88
Guiyang	146 (1.38)	0.075	0.058	0.027	75.5	14.77	77.48	81.50
Kunming	104 (0.97)	0.070	0.041	0.046	66.0	16.63	59.79	181.16
Lhasa	21 (0.42)	0.049	0.008	0.021	32.0	10.14	29.33	265.81
Xi'an	219 (1.83)	0.120	0.046	0.046	63.0	14.83	44.57	147.91
Lanzhou	253 (2.39)	0.153	0.058	0.046	54.5	7.95	15.75	209.50
Xining	62 (1.01)	0.133	0.041	0.029	56.5	6.28	36.00	218.45
Yinchuan	29 (0.66)	0.092	0.042	0.029	49.5	10.38	16.10	232.91
Urumqi	121 (1.01)	0.137	0.091	0.068	56.0	7.70	26.48	235.45
Baoji	467 (4.36)	0.024	0.028	0.106	63.0	14.05	61.97	137.53
Yuxi	96 (0.59)	0.082	0.059	0.022	76.0	17.15	52.83	201.37
Yichang	286 (2.72)	0.086	0.046	0.025	75.5	17.35	105.99	101.83
Shenzhen	117 (1.63)	0.057	0.012	0.044	71.5	23.10	135.21	156.79
Zhongshan	139 (1.34)	0.052	0.029	0.040	79.0	22.60	165.94	153.77
Qingdao	337 (3.35)	0.099	0.052	0.046	63.8	13.00	57.47	183.21
Yantai	623 (4.74)	0.082	0.043	0.040	67.5	12.40	59.45	204.75
Linyi	60 (0.60)	0.112	0.082	0.049	63.6	13.84	58.01	185.46
Suzhou	352 (3.22)	0.090	0.034	0.052	69.0	17.20	89.10	149.67
Anqing	272 (2.97)	0.086	0.053	0.038	72.5	17.25	130.68	136.30
Wenzhou	250 (2.54)	0.081	0.028	0.056	73.5	18.70	163.53	126.50
Xiamen	236 (2.13)	0.063	0.021	0.043	74.0	21.10	130.70	164.96

*Note*. PM10: particulates with an aerodynamic diameter of 10 mm; SO_2_: sulfur dioxide; NO_2_: nitrogen dioxide.

**Table 3:** Descriptive statistical analysis of the variables.

Variables	*N*	Minimum	Maximum	Mean	Std. deviation
Asthma prevalence	43	0.420	5.730	2.252	1.297
PM10	43	0.024	0.153	0.091	0.026
SO_2_	43	0.007	0.091	0.042	0.018
NO_2_	43	0.016	0.106	0.042	0.015
Relative humidity	43	32.000	81.000	66.008	10.366
Air temperature	43	4.750	24.440	15.125	4.994
Precipitation	43	15.750	211.390	82.924	46.516
Hours of sunshine	43	67.880	265.810	166.087	43.524

*Note*. PM10: particulates with an aerodynamic diameter of 10 mm; SO_2_: sulfur dioxide; NO_2_: nitrogen dioxide.

**Table 4:** The relationships among air pollutants, climatic factors, and asthma prevalence by Pearson correlation coefficient.

Variables	Asthma prevalence	PM10	SO_2_	NO_2_	Relative humidity	Air temperature	Precipitation	Hours of sunshine
Asthma prevalence	1	−0.101	−0.323^*∗*^	0.261	0.351^*∗*^	0.289	0.273	−0.476^*∗∗*^
PM10		1	0.607^*∗∗*^	−0.023	−0.308^*∗*^	−0.551^*∗∗*^	−0.529^*∗∗*^	0.231
SO_2_			1	0.050	−0.213	−0.480^*∗∗*^	−0.500^*∗∗*^	0.225
NO_2_				1	0.029	−0.061	−0.037	−0.157
Relative humidity					1	0.668^*∗∗*^	0.740^*∗∗*^	−0.771^*∗∗*^
Air temperature						1	0.821^*∗∗*^	−0.635^*∗∗*^
Precipitation							1	−0.577^*∗∗*^
Hours of sunshine								1

*Note*. PM10: particulates with an aerodynamic diameter of 10 mm; SO_2_: sulfur dioxide; NO_2_: nitrogen dioxide. ^*∗*^ *p* < 0.05. ^*∗∗*^ *p *< 0.01.

**Table 5:** Multiple linear regression analysis of risk factors and asthma prevalence in children.

Model	Unstandardized coefficients		Standardized coefficients	*t*	Sig.	Collinearity statistics	
	B	Std. error	Beta			Tolerance	VIF
Constant	1.673	3.104		0.539	0.593		
SO_2_	−19.572	9.654	−0.271	−2.027	0.050	0.930	1.075
Relative humidity	−0.036	0.028	−0.291	−1.294	0.204	0.329	3.041
Hours of sunshine	−0.014	0.006	−0.458	−2.256	0.030	0.402	2.489

*Note*. PM10: particulates with an aerodynamic diameter of 10 mm; SO_2_: sulfur dioxide; predictors: SO_2_, relative humidity, and hours of sunshine; dependent variable: asthma prevalence; *R* ^2^ = squared multiple correlation of *y* with *x*. *R* ^2^ = 0.303, *F* = 5.573, and *p* < 0.01.
